# Optimizing Aortic Valve Replacement Through Strategic Upsizing: A Modern Framework for Lifetime Valve Management

**DOI:** 10.3390/diseases14030103

**Published:** 2026-03-12

**Authors:** Dimitrios E. Magouliotis, Vasiliki Androutsopoulou, Andrew Xanthopoulos, Noah Sicouri, Bo Yang

**Affiliations:** 1 Department of Cardiac Surgery Research, Lankenau Institute for Medical Research, Wynnewood, PA 19096, USA; 2 Department of Cardiothoracic Surgery, University of Thessaly, Biopolis, 41110 Larissa, Greece; androutsopoulouvasiliki@uth.gr; 3 Department of Cardiology, University of Thessaly, Biopolis, 41110 Larissa, Greece; andrewvxanth@gmail.com; 4 Department of Neuroscience, University of Pittsburgh, Pittsburgh, PA 15260, USA; nps67@pitt.edu; 5 Department of Cardiac Surgery, University of Michigan, Ann Arbor, MI 48109, USA; boya@med.umich.edu

**Keywords:** aortic annular enlargement, prosthesis–patient mismatch, aortic valve replacement, valve-in-valve TAVR, lifetime valve management

## Abstract

Aortic valve disease is increasingly recognized as a chronic, progressive condition in which the initial valve intervention exerts a decisive influence on all subsequent therapeutic options. The persistence of prosthesis–patient mismatch (PPM), often driven by implantation of small surgical prostheses (≤21–23 mm), is associated with higher residual transvalvular gradients, attenuated left ventricular reverse remodeling, inferior long-term survival, and compromised outcomes following valve-in-valve (ViV) transcatheter procedures. Accumulating clinical and imaging evidence indicates that aortic annular enlargement (AAE), particularly using contemporary Y-incision and extended “roof” reconstruction techniques, can safely and reproducibly expand the annulus, sinuses of Valsalva, and sinotubular junction, thereby permitting implantation of larger prostheses and substantially reducing the risk of PPM. Insights from computational fluid dynamics further demonstrate that annular and root enlargement favorably alters postoperative flow dynamics, resulting in lower peak velocities, reduced pressure gradients, and more physiologic flow patterns in both primary surgical valve replacement and simulated ViV settings. From a lifetime management perspective, valve diameter optimization emerges as a critical determinant of both immediate hemodynamic performance and future procedural feasibility. Surgical programs that adopt a systematic approach to anatomic assessment, valve sizing strategy, PPM surveillance, and ViV preparedness may achieve meaningful improvements in short- and long-term outcomes. This review integrates anatomic, operative, hemodynamic, and quality-oriented evidence to support consideration of valve upsizing as a central principle in contemporary aortic valve replacement.

## 1. Introduction

Aortic valve disease is increasingly managed as a long-term, iterative condition rather than a problem resolved by a single intervention [1]. Improvements in surgical and transcatheter therapies, coupled with increased life expectancy, mean that many patients now undergo multiple valve procedures over the course of their lifetime, progressing through combinations of surgical aortic valve replacement (SAVR), transcatheter aortic valve replacement (TAVR), valve-in-valve (ViV) interventions, and, in selected cases, redo-SAVR [1]. As a result, contemporary treatment paradigms have shifted beyond the immediate procedural outcome of the index operation to encompass the durability, hemodynamic performance, and feasibility of all future interventions. This evolution in thinking is further supported by recent analyses of aortic root and annular enlargement strategies. In particular, the latest Society of Thoracic Surgeons review demonstrates that aortic root enlargement (ARE) can be performed safely and reproducibly, achieving predictable enlargement of one to three valve sizes without an associated increase in operative mortality when undertaken in experienced, high-volume centers [2]. Collectively, these data challenge the notion that annular or root enlargement should be reserved for exceptional circumstances and instead position these techniques as integral components of modern surgical planning with direct implications for long-term hemodynamics and future transcatheter options.

Within this lifetime-oriented framework, prosthesis–patient mismatch (PPM) has emerged as a key determinant of long-term outcome. Originally described by Rahimtoola in 1978 as an “iatrogenic stenosis” resulting from implantation of a prosthetic valve with an effective orifice area disproportionate to patient body size [3], PPM remains highly prevalent following SAVR. A substantial body of evidence, including seminal work by Pibarot, Dumesnil, and others, has consistently linked PPM to elevated residual gradients, impaired left ventricular (LV) mass regression, increased heart failure (HF) symptoms, and reduced long-term survival [1,4,5,6,7]. Importantly, the adverse impact of PPM is not confined to surgical therapy; registry analyses have demonstrated that severe PPM following TAVR is similarly associated with higher mortality and increased rates of HF rehospitalization [8,9].

The geometric configuration established at the time of the initial SAVR plays a decisive role in shaping these outcomes. The annular diameter, sinus dimensions, and sinotubular junction (STJ) geometry created during the index operation effectively define the fixed anatomy of the aortic root, as these structures cannot be substantially modified by subsequent transcatheter interventions. This anatomical “lock-in” governs the feasibility and safety of future procedures, particularly ViV TAVR, by influencing achievable post-procedural gradients and the risk of coronary obstruction. Accordingly, as highlighted by Errico and Hui, aortic annular enlargement (AAE) and deliberate valve upsizing should be conceptualized as anticipatory strategies within lifetime valve management, rather than as reactive measures reserved solely for patients with an overtly small native annulus [10].

Against this background, the present review explores whether valve diameter optimization—achieved through systematic application of annular or root enlargement techniques—may represent a fundamental quality metric for contemporary SAVR programs. We synthesize pathophysiological insights, clinical outcome data, advances in Y-incision and extended “roof” enlargement techniques, evidence from advanced imaging and computational fluid dynamics (CFD), and emerging findings from statewide quality improvement (QI) initiatives to support a unified, lifetime-oriented rationale for valve upsizing in modern aortic valve replacement. Consistent with this shift, the 2025 ESC/EACTS guidelines emphasize prevention of prosthesis-patient mismatch whenever possible and explicitly note SAVR with aortic root enlargement (or use of a supra-annular transcatheter valve platform) as strategies in patients with a small annulus who are at risk of severe PPM based on predicted effective orifice area [1]. While individual components of prosthesis-patient mismatch, annular enlargement techniques, and valve-in-valve considerations have been previously described, these elements are often discussed in isolation. The present review integrates anatomic reconstruction, hemodynamic modeling, procedural durability, and system-level quality improvement into a unified lifetime management framework centered on valve diameter optimization. This integrative perspective (linking operative geometry to downstream transcatheter feasibility and measurable quality metrics) represents the principal conceptual contribution of this work.

## 2. Prosthesis–Patient Mismatch and the Consequences of Small Prosthetic Valves

Prosthesis–patient mismatch is conventionally characterized by an indexed effective orifice area (iEOA) that is inadequate for a patient’s body surface area, resulting in persistently elevated transvalvular gradients despite a normally functioning prosthetic valve. Thresholds commonly used to define clinically relevant mismatch include an iEOA ≤ 0.65 cm^2^/m^2^ for severe PPM and 0.65–0.85 cm^2^/m^2^ for moderate PPM. Across multiple surgical series, both degrees of mismatch have been consistently linked to higher residual gradients and attenuated left ventricular (LV) reverse remodeling following SAVR [5,6,7]. Large-scale registry studies and meta-analyses further demonstrate that the presence of PPM is associated with increased long-term all-cause and cardiac mortality [6,7,11].

The adverse clinical impact of PPM is particularly evident in patients with longer anticipated survival. Analyses by Head et al. showed that PPM confers an excess risk of late mortality in recipients of both mechanical and bioprosthetic valves, with the magnitude of this effect being greatest among younger patients and those with impaired LV function [6]. Similarly, Fallon and colleagues reported that PPM remains prevalent in contemporary SAVR practice and independently predicts inferior long-term survival, despite advances in prosthetic valve design and sizing methodologies [7]. More recent pooled analyses support these observations, suggesting that even moderate degrees of mismatch may exert clinically meaningful effects over extended follow-up periods [11].

Of note, the introduction of transcatheter aortic valve replacement has not eliminated the problem of PPM. Data from the STS/ACC Transcatheter Valve Therapy (TVT) registry indicate that severe PPM occurs in approximately 12% of patients undergoing TAVR and is associated with higher one-year mortality and increased rates of HF rehospitalization [8,9]. Although supra-annular transcatheter valve platforms reduce the incidence of PPM relative to intra-annular designs, mismatch remains frequent in patients with small native annuli and cannot be reliably avoided by device selection alone [9].

Beyond its immediate hemodynamic implications, implantation of a small surgical prosthesis may compromise future reintervention strategies. Reduced internal valve diameter is a major determinant of elevated gradients after ViV TAVR and increases the risk of neo-PPM, sinus sequestration, and coronary obstruction. Computed tomography–based morphological studies have identified small sinotubular junction (STJ) diameters, limited sinus volumes, and unfavorable coronary heights as key anatomical predictors of ViV-related complications and diminished durability of subsequent interventions [10,12]. Consequently, selection of a small valve at the index operation can adversely affect both early postoperative performance and long-term procedural flexibility.

In this setting, comparative data underscore the hemodynamic advantages of surgical enlargement strategies over transcatheter therapy in anatomically constrained aortic roots. Using patient-specific computational modeling combined with matched clinical analyses, Monaghan and colleagues demonstrated that Y-incision annular enlargement yields significantly lower postoperative gradients and more favorable outflow characteristics than TAVR in small-root anatomy, while simultaneously creating geometry better suited for future ViV procedures [13]. These findings support the concept that deliberate valve upsizing during SAVR provides hemodynamic and anatomic benefits that cannot be consistently replicated by transcatheter approaches alone in patients with small annuli. The clinical, hemodynamic, and lifetime-management implications of implanting a small versus an upsized prosthesis are summarized in Table 1.

Recent comparative evidence further contextualizes the role of SAVR with enlargement versus TAVR in patients with small aortic annuli. In the randomized VIVA trial (151 patients ≥65 years with severe aortic stenosis and CT-defined small annulus), TAVR and SAVR achieved similar early clinical outcomes, with comparable rates of the composite endpoint of impaired valve hemodynamics (severe PPM or moderate–severe aortic regurgitation) at 60 days [22]. However, broader syntheses suggest clinically relevant trade-offs: contemporary meta-analyses indicate broadly similar short-term mortality between TAVR and SAVR in small annulus cohorts, while highlighting that TAVR may carry higher risks of moderate/severe paravalvular regurgitation and permanent pacemaker implantation in this anatomic subgroup—factors that may be particularly important in lifetime management planning. Accordingly, in operable patients with small annuli, a strategy of SAVR combined with annular/root enlargement remains a key option to minimize PPM and preserve valve-in-valve feasibility without accepting the downstream limitations of a small surgical prosthesis [22,23,24].

It is important to recognize that much of the evidence supporting valve upsizing and annular enlargement derives from observational registries, single-center surgical series, and computational or modeling analyses. While these studies consistently demonstrate favorable hemodynamic profiles and biological plausibility, randomized multicenter trials specifically evaluating annular enlargement strategies and long-term survival remain limited. Accordingly, the conclusions of this review should be interpreted within the context of evolving evidence rather than definitive practice mandates.

## 3. Aortic Annular Enlargement and Valve Upsizing: Safety Profile and Clinical Impact

Aortic annular and root enlargement techniques, including classical approaches such as the Nicks, Manouguian, and Konno procedures and their modifications, have been described for several decades. Nevertheless, concerns related to operative complexity, bleeding risk, and prolonged cardiopulmonary bypass or cross-clamp times have historically limited their widespread adoption in routine surgical practice. In recent years, however, a growing body of contemporary evidence has challenged these perceptions. In a meta-analysis evaluating outcomes of aortic root enlargement in patients undergoing SAVR, Yu and colleagues demonstrated that annular or root enlargement is not associated with increased early mortality or major perioperative complications and is effective in significantly reducing the incidence of PPM [25]. Of note, the authors highlighted that enlargement strategies facilitate implantation of larger prosthetic valves at the index operation, a consideration of increasing relevance in the era of transcatheter ViV therapies [25].

These findings are supported by additional systematic evaluations. Tanaka et al. reported that early and mid-term outcomes following aortic annular enlargement are comparable to those achieved with isolated SAVR when procedures are performed in experienced centers, while offering superior postoperative hemodynamics and lower rates of PPM [26]. In parallel, Fazmin and colleagues, in a recent narrative review, characterized annular enlargement as an underutilized yet effective intervention for minimizing PPM and aligning surgical strategy with contemporary lifetime valve management principles [27].

Observational data from single-center experiences further reinforce the safety and durability of these approaches. For example, Okamoto and co-workers reported favorable early and late outcomes following Nicks annular enlargement in elderly patients, without an associated increase in operative mortality compared with standard SAVR [10,26]. Other institutional series have similarly demonstrated stable long-term survival and low reoperation rates after aortic root enlargement, suggesting that when techniques are standardized and performed by experienced teams, the incremental operative risk remains modest [26,27].

Collectively, the available evidence suggests that annular and root enlargement may not be associated with substantially increased operative risk when performed in experienced centers. In appropriately selected patients, aortic annular enlargement enables implantation of larger prostheses, reduces the likelihood of PPM, improves postoperative hemodynamic performance, and can be achieved without a meaningful increase in early mortality or major morbidity when performed in centers with sufficient expertise [25,26,27]. These observations support a conceptual shift in which annular enlargement is framed not as an exceptional maneuver, but as a deliberate, quality-oriented strategy within modern SAVR practice.

## 4. Y-Incision and “Roof” Techniques: Expanding the Functional Limits of Annular Enlargement

Conventional AAE techniques are typically associated with limited degrees of upsizing, most often permitting expansion by one to two prosthetic valve sizes. The development of the Y-incision with rectangular patch reconstruction, frequently referred to as the Yang technique, has substantially altered this paradigm by enabling a far greater degree of annular and root expansion. Initially described by Yang and colleagues, this approach involves a Y-shaped incision across the aortomitral curtain followed by patch reconstruction, allowing predictable enlargement of the annulus by three to four valve sizes [28]. Subsequent refinements have further extended the capacity of this strategy. In particular, incorporation of a modified “roof” aortotomy closure has been shown to expand not only the annulus but also the sinotubular junction and proximal ascending aorta, facilitating upsizing by up to five valve sizes even in patients with markedly small native annuli (≤17 mm) [16,17]. The surgical steps of the Y-incision aortic annular enlargement and the associated “roof” modification have been described in detail in prior technical reports and operative illustrations by Yang and colleagues; therefore, they are not reproduced here and readers are referred to the original publications for comprehensive procedural visualization [16,17,28,29]. Importantly, these techniques have expanded the conceptual boundaries of annular enlargement by demonstrating that substantial valve upsizing can be achieved with reproducible surgical reconstruction, thereby enabling a lifetime-oriented strategy for aortic valve management.

The mechanistic distinction between classical annular enlargement techniques and the Y-incision approach lies in the geometry and distribution of expansion. Traditional techniques such as the Nicks and Manouguian procedures primarily extend posteriorly through the non-coronary sinus and may partially involve the aortomitral curtain, resulting in asymmetric enlargement that is often constrained by residual sinotubular junction (STJ) geometry [25,26,27]. Consequently, these approaches typically permit upsizing by one to two valve sizes. In contrast, the Y-incision technique distributes expansion across the aortomitral curtain using a rectangular patch configuration that widens the basal ring more symmetrically [16,28,30]. This configuration enables predictable enlargement of three to four valve sizes in contemporary series [16,18]. When combined with a roof extension, the approach further enlarges the STJ and proximal ascending aorta, thereby addressing supravalvular restriction in addition to annular constraint [17,31]. Computed tomography analyses confirm that Y-incision with roof modification produces coordinated expansion of the annulus, sinuses of Valsalva, and STJ, resulting in comprehensive three-dimensional root remodeling rather than localized posterior dilation [15,31].

Historically, limitations of classical posterior enlargement methods have included incomplete basal ring expansion, persistent supravalvular restriction, and limited reproducibility in achieving substantial upsizing [25,26]. Because STJ geometry remains largely unmodified in traditional techniques, residual supravalvular constraint may limit the achievable internal prosthesis diameter even when posterior annular expansion is performed. In contrast, standardized Y-incision strategies aim to overcome these constraints by redistributing tension across the aortomitral curtain and, when necessary, incorporating roof extension to expand the STJ, thereby enabling greater and more reproducible valve upsizing [14,16,17,31].

Clinical experience has demonstrated that the applicability of Y-incision enlargement extends beyond isolated SAVR. Cangut and colleagues reported favorable outcomes when Y-incision AAE was performed in conjunction with mitral valve surgery, achieving consistent valve upsizing, low postoperative gradients, and no increase in perioperative complications despite the added procedural complexity [29]. These findings indicate that the technique can be safely integrated into combined valve operations when lifetime optimization of aortic valve geometry is a priority. Early institutional experience from the University of Michigan further supports the feasibility of this approach. In that series, patients with a median native annular diameter of approximately 21 mm were able to receive prostheses measuring 27–29 mm, with acceptable operative mortality and complication rates [18,19]. Within this program, Y-incision enlargement has become the preferred method for annular and root enlargement, owing to its ability to preserve mitral valve integrity, accommodate implantation of larger prostheses, expand sinus and STJ dimensions, and reconstruct the aortic root into a more cylindrical configuration favorable for future ViV transcatheter interventions [16,29].

Comparative outcome data reinforce these observations. In a propensity-matched analysis of 380 patients, Makkinejad and colleagues compared Y-incision AAE with traditional enlargement techniques, including Nicks and Manouguian procedures [14]. In the matched cohort, the Y-incision group achieved a substantially larger median prosthesis size (27 mm versus 23 mm) without an increase in operative mortality or major perioperative complications [14]. These findings suggest that Y-incision enlargement offers a greater degree of annular expansion while maintaining a safety profile comparable to conventional AAE strategies.

More granular outcome data from contemporary Y-incision series further clarify the safety profile of this approach. In the Michigan experience, early operative mortality has been reported in the low single-digit range and comparable to isolated SAVR, despite substantial annular upsizing [14,18]. Rates of permanent pacemaker implantation, stroke, and reoperation for bleeding have similarly not demonstrated significant excess relative to matched cohorts undergoing traditional enlargement or isolated SAVR [14]. Importantly, paravalvular leak is uncommon when rectangular patch reconstruction is performed meticulously, as the technique allows controlled geometric expansion rather than irregular annular distortion [16,19]. Reports of coronary injury are rare, and careful respect of coronary ostial height and sinus geometry is emphasized in operative planning [15,32]. Collectively, these data suggest that, when performed in experienced centers, Y-incision enlargement achieves substantial annular expansion without introducing disproportionate rates of conduction disturbance, coronary compromise, or structural instability.

Beyond operative outcomes, the anatomical and hemodynamic consequences of Y-incision enlargement have been systematically characterized. Using pre- and postoperative computed tomography, Truesdell and colleagues demonstrated significant increases in sinus dimensions and STJ diameter following Y-incision AAE, with or without roof extension, confirming that the technique results in comprehensive reconfiguration of the aortic root rather than isolated annular stretching [15]. The adaptability of the approach to complex coronary anatomy has also been demonstrated. Yin and co-workers reported successful application of Y-incision enlargement in patients with an anomalous left circumflex artery arising from the right coronary sinus, achieving meaningful enlargement while preserving coronary integrity through tailored patch geometry and careful respect of inter-arterial planes [32]. Additional work by Wang et al. showed that combining the Y-incision with an updated roof technique produces uniform expansion of both the aortic root and STJ, creating geometry that is particularly favorable for subsequent ViV procedures [31].

Computational fluid dynamics (CFD) modeling provides insight beyond standard Doppler echocardiography by enabling three-dimensional visualization of velocity vectors, wall shear stress distribution, turbulent kinetic energy, and blood residence time within patient-specific aortic root geometries [20,21,30,31]. While Doppler echocardiography accurately measures peak velocity and mean transvalvular gradients, it cannot directly quantify spatial flow patterns, recirculation zones, or localized shear stress gradients that may influence long-term prosthetic durability and thrombogenic potential. In CFD analyses of Y-incision enlargement, reductions in peak systolic velocity and transvalvular gradient were accompanied by decreased turbulent kinetic energy and more uniform flow distribution across the prosthetic valve [20]. Blood residence time (defined as the duration that blood elements remain within a defined flow domain) serves as a surrogate marker for stagnation and thrombosis risk in low-flow regions. CFD simulations have demonstrated that Y-incision enlargement does not increase pathologic residence time or flow stagnation despite substantial geometric remodeling [20]. Although clinical thrombosis attributable specifically to annular enlargement has not been reported as a prevalent complication, the absence of increased flow stagnation in modeling studies provides mechanistic reassurance regarding hemodynamic safety. Importantly, the potential future role of CFD lies in preoperative planning. Patient-specific CT-based simulations may allow surgeons to model projected valve size, root geometry, and anticipated valve-in-valve configurations, thereby optimizing prosthesis selection and extent of enlargement before entering the operating room [31,33,34].

Taken together, these data indicate that Y-incision and extended roof techniques transform annular enlargement from a limited, incremental maneuver into a comprehensive reconstructive strategy. By enabling alignment of prosthetic valve size with patient-specific annular anatomy, reducing the risk of PPM, and creating a robust anatomic platform for future ViV TAVR, these approaches represent a pivotal advance in surgical lifetime management of aortic valve disease. The principal anatomical and hemodynamic effects associated with Y-incision and roof enlargement techniques are summarized in Table 2. The technical and geometric distinctions between classical posterior enlargement procedures and contemporary Y-incision strategies are summarized in Table 3.

## 5. Valve Diameter Optimization as a Quantifiable Quality Indicator

For a parameter to function as a meaningful quality-improvement (QI) indicator, it must be clearly defined, objectively measurable, and demonstrably linked to outcomes that matter to patients. Within this framework, optimization of prosthetic valve diameter can be operationalized using several complementary process and outcome measures. Rather than representing a single binary decision, valve upsizing reflects a spectrum of practice patterns that can be systematically evaluated at the program level.

One approach involves monitoring the distribution of prosthesis sizes implanted, particularly among patients with small-to-normal annular dimensions as defined by preoperative computed tomography (for example, annular diameters of 19–25 mm). In a quality-oriented surgical practice, implantation of very small valves (≤21 mm) should be uncommon in patients with normal body size, while a substantial proportion of cases should achieve alignment between prosthetic internal diameter and the CT-derived basal ring dimension [10,18,30]. Tracking these distributions over time provides a practical means of identifying unwarranted variation in valve sizing strategies.

A second, complementary measure is the utilization rate of aortic annular or root enlargement (AAE/ARE) among patients deemed anatomically eligible and at elevated risk for PPM. Despite robust evidence supporting the safety and efficacy of annular enlargement, multiple meta-analyses and contemporary reviews indicate that these techniques remain underused in routine SAVR practice [25,26,27]. Importantly, the objective of a mature QI program is not indiscriminate application of AAE, but rather consistent and deliberate consideration of enlargement strategies in patients for whom the risk of PPM is moderate or high.

Outcome-based assessment provides a third dimension for evaluating valve sizing quality. The prevalence of moderate and severe PPM following SAVR represents a clinically meaningful endpoint that can be readily tracked at the institutional level. Large registry analyses continue to demonstrate that PPM remains common and prognostically relevant despite advances in prosthetic valve design [6,7,11]. In addition to registry-level observations, multiple large-scale meta-analyses have demonstrated that moderate-to-severe prosthesis–patient mismatch is independently associated with reduced long-term survival and impaired left ventricular mass regression following SAVR [35,36,37]. These findings reinforce the concept that prosthesis size is not merely a technical variable but a determinant of durable hemodynamic performance and long-term clinical trajectory. Programs that systematically pursue valve upsizing and appropriately employ annular enlargement would be expected to demonstrate a progressive reduction in PPM rates over time.

**Figure 1:**
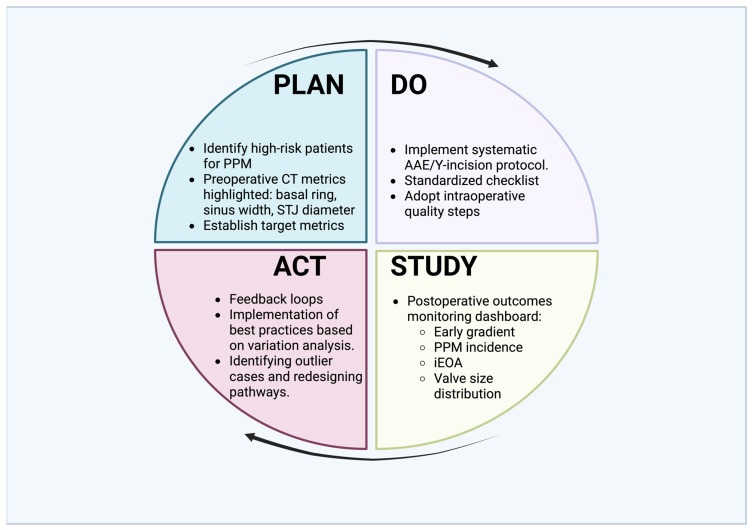
Plan–Do–Study–Act (PDSA) framework for valve upsizing in surgical aortic valve replacement. The diagram depicts an iterative quality-improvement cycle integrating aortic annular enlargement (AAE) into routine practice. Plan: Identify patients at risk for prosthesis–patient mismatch (PPM) using preoperative CT metrics (basal ring, sinus width, sinotubular junction). Do: Apply standardized AAE/Y-incision protocols. Study: Track postoperative gradients, indexed effective orifice area (iEOA), PPM rates, and valve size distribution. Act: Use feedback to reduce variation, refine pathways, and implement best practices. This approach positions valve upsizing as a measurable quality metric aligned with lifetime aortic valve management. This schematic emphasizes the transition from isolated procedural decision-making to structured lifetime management planning.

These individual measures can be integrated into a composite valve sizing quality index that aligns surgical decision-making with lifetime management objectives. One example is the proportion of patients younger than 70 years in whom (a) the implanted prosthesis internal diameter falls within 1–2 mm of the CT-derived basal ring diameter and (b) moderate or severe PPM is absent on early postoperative echocardiography. Such a composite metric links a technical strategy (annular enlargement and valve upsizing) to its immediate functional consequence (optimized hemodynamics) and its anticipated long-term impact, including the feasibility of future ViV interventions [10,14,18,26,27]. International registry data on valve-in-valve procedures demonstrate that initial surgical prosthesis size substantially influences subsequent transcatheter feasibility and residual gradients, underscoring the importance of forward-planning during the index operation [38,39]. Contemporary quality assessment in cardiac surgery increasingly incorporates process-based and composite performance measures derived from large national databases rather than relying solely on perioperative mortality [40,41]. Within this broader framework, prosthesis size optimization represents a measurable structural parameter that can be integrated into institutional benchmarking strategies. Figure 1 illustrates how these elements may be incorporated into a structured Plan–Do–Study–Act (PDSA) framework to support continuous improvement in aortic valve replacement practice.

Importantly, the conceptualization of valve upsizing as a quality indicator should not be interpreted as a mandate for universal annular enlargement. Quality metrics must account for patient-specific risk, anatomical feasibility, comorbidity burden, and life expectancy. In frail patients, those with prohibitive operative risk, or individuals with limited anticipated survival, the incremental complexity of enlargement may not confer meaningful clinical advantage. Formal frailty assessment and individualized risk stratification have emerged as critical components of surgical decision-making in older adults undergoing cardiac procedures [42,43]. Incorporating these variables into enlargement strategy selection ensures that valve diameter optimization remains aligned with patient-centered outcomes rather than procedural uniformity. Accordingly, valve diameter optimization should be framed as a structured consideration within shared decision-making rather than an obligatory procedural endpoint.

## 6. Lifetime Management Pathways and the Strategic Importance of Valve Upsizing

Effective lifetime management of aortic valve disease requires individualized stratification based on patient age, comorbidity burden, valve morphology, and the anticipated likelihood of future valve-related interventions. Several decision-making frameworks, including those proposed by Medranda and colleagues, have emphasized that the choice between transcatheter and surgical strategies at the index intervention directly influences the feasibility and quality of subsequent treatment options [33]. Within these paradigms, AAE and deliberate valve upsizing represent enabling strategies that align the initial operation with long-term therapeutic planning.

In younger and middle-aged patients, typically defined as those younger than 70–75 years, who undergo bioprosthetic SAVR, the probability of at least one future reintervention is substantial. This expectation is reinforced by contemporary guideline discussions and long-term durability series demonstrating that structural valve deterioration accelerates in younger patients, making reintervention planning central to lifetime strategy selection [44,45]. For this population, the primary objective of the initial operation extends beyond immediate valve replacement to the creation of an anatomic platform that supports future ViV transcatheter procedures with acceptable residual gradients and a low risk of coronary obstruction. Large international registries of valve-in-valve therapy highlight that smaller index surgical prostheses are associated with higher residual gradients and that coronary obstruction, while uncommon, remains a high-consequence complication driven by valve–coronary relationships that can be assessed using CT-based planning [46,47]. In high-risk anatomies, adjunctive transcatheter strategies such as intentional leaflet laceration (BASILICA) have been developed to mitigate coronary obstruction and expand the feasibility of redo transcatheter interventions [48]. Achieving a prosthesis internal diameter that closely approximates the CT-derived basal ring dimension is therefore critical, a goal that frequently necessitates Y-incision AAE even in patients whose native annulus would not traditionally be considered small [14,18,30]. This consideration is particularly relevant in bicuspid aortic valve disease, where asymmetric root geometry and associated aortic dilation may further constrain future transcatheter options and heighten the importance of root enlargement and valve upsizing.

In older patients with more limited life expectancy, decision-making is inherently more nuanced. Nonetheless, available data indicate that annular enlargement can be performed safely even in octogenarians with small annuli and may offer symptomatic benefit through reduction in transvalvular gradients and mitigation of PPM [26]. For physiologically robust elderly patients, especially those with an anticipated survival exceeding a decade, implantation of a very small prosthesis may still represent a suboptimal lifetime strategy, as it restricts ViV feasibility and increases the likelihood of redo surgery or recurrent heart failure. In selected failed surgical bioprostheses, bioprosthetic valve fracture has been used to increase the effective internal diameter and improve post–valve-in-valve gradients, further underscoring how the index prosthesis design and size influence downstream transcatheter options [49].

The relevance of valve upsizing becomes even more apparent in complex or redo settings, including TAVR explantation, prosthetic valve endocarditis, and SAVR following failed ViV interventions. In these scenarios, Y-incision enlargement and related root reconstruction techniques can restore otherwise unfavorable anatomy, permitting implantation of a larger prosthesis and effectively resetting the patient’s lifetime management trajectory [15,20,30,31,32]. This principle has become increasingly relevant in the contemporary era of TAVR explantation, where international registry data demonstrate that surgical explant is associated with non-trivial morbidity and mortality, reinforcing the value of optimizing anatomy and prosthesis size at the initial operation whenever feasible [50]. However, the elevated risk associated with such reoperative contexts underscores a broader principle: optimal anatomic configuration is most safely and effectively achieved at the index operation, when tissue planes are preserved and operative risk is lower.

Across patient subsets and clinical scenarios, a consistent theme emerges—prosthetic valve diameter is not a neutral technical choice, but a determinant that shapes long-term hemodynamics, reintervention options, and overall disease trajectory. Systematic valve upsizing, supported by contemporary annular enlargement techniques when appropriate, reflects a proactive and lifetime-oriented approach to quality in aortic valve surgery. It is important to distinguish between association and causation in this context. While prosthesis-patient mismatch has consistently been associated with adverse long-term outcomes, and annular enlargement reduces the incidence of PPM, direct evidence demonstrating that enlargement itself independently improves long-term survival remains limited. Most available data derive from observational analyses, and randomized trials specifically evaluating enlargement strategies with long-term follow-up have not yet been performed. Therefore, valve upsizing should be interpreted as a strategy supported by strong physiological rationale and consistent associative data, rather than definitive survival-level evidence.

Despite the advantages of annular enlargement in selected patients, it is neither universally necessary nor universally feasible. Patients with advanced frailty, prohibitive operative risk, severe ventricular dysfunction, or limited life expectancy may derive limited benefit from incremental increases in prosthesis diameter relative to the additional operative complexity required [25,26,27]. Frailty has been consistently associated with worse outcomes after both SAVR and TAVR, and structured frailty assessment has been proposed as an important adjunct to conventional risk models when tailoring invasive valve strategies [51]. Similarly, in heavily calcified roots, prior radiation, destructive endocarditis, or complex reoperative settings, extensive enlargement may carry disproportionate technical risk. From an anatomic perspective, there is no absolute lower annular size below which enlargement is impossible; however, the achievable degree of expansion depends on tissue quality, integrity of the aortomitral curtain, and the surgeon’s experience. Contemporary Y-incision series have demonstrated successful upsizing even in annuli measuring ≤17 mm [16,17], yet such expansion requires careful reconstruction and may not be appropriate in all operative contexts. Therefore, annular enlargement should be considered a selective reconstructive strategy rather than a universal procedural mandate, guided by patient-specific anatomy, operative risk, and lifetime treatment goals.

## 7. System-Level Quality Improvement: Valve Upsizing as an Organizational Strategy

Although single-center experiences provide important proof of concept, sustained transformation in surgical practice typically requires coordinated, system-level approaches. Statewide quality collaboratives offer a unique platform for implementing and evaluating such strategies at scale. The Michigan Society of Thoracic and Cardiovascular Surgeons Quality Collaborative (MSTCVS-QC) has been widely recognized for its data-driven efforts to improve cardiac surgical outcomes across institutions. Within this framework, a recent statewide initiative focused specifically on increasing adoption of AAE and valve upsizing during SAVR [34].

Using Society of Thoracic Surgeons (STS) database data to characterize baseline practice patterns, the initiative examined SAVR cases performed with and without annular enlargement and incorporated targeted feedback on prosthesis size distribution and prosthesis–patient mismatch rates. Educational efforts addressing contemporary enlargement techniques, including Y-incision approaches, were combined with consensus-building around lifetime valve management principles [34]. Following implementation, a substantial shift in practice was observed, with the proportion of SAVR cases incorporating AAE increasing from approximately 7% to nearly 20%. This change was accompanied by an increase in median prosthesis size and broader uptake of Y-incision techniques among participating surgeons, without an associated increase in early mortality or major perioperative complications [34]. These findings reinforce the concept that annular enlargement and valve upsizing can be safely expanded at the population level when supported by structured quality initiatives.

Beyond its immediate procedural effects, this statewide experience illustrates how valve diameter optimization can be operationalized as an explicit quality-improvement objective. By benchmarking rates of small valve implantation, AAE utilization, and PPM incidence against collaborative norms, institutions can identify practice variation and establish targeted improvement goals. Educational strategies (including proctoring, simulation-based training, and peer-to-peer mentorship) may further reduce perceived technical barriers to adoption of advanced enlargement techniques [13,14,26,30,34]. Table 4 summarizes example metrics that can be used by individual centers or regional collaboratives to monitor performance and assess the impact of valve upsizing strategies over time.

Within a broader QI framework, valve upsizing can also be linked to downstream clinical outcomes such as heart failure readmissions, postoperative echocardiographic gradients, and long-term reintervention rates. This creates a feedback loop in which optimization of aortic root geometry at the index operation translates into measurable improvements in patient-centered outcomes across the continuum of aortic valve disease management.

Beyond the technical feasibility of annular and root enlargement, the scalability of a “valve-upsizing–first” paradigm depends on implementation strategy. Regional collaboratives have demonstrated that structured audit, benchmarking, and shared learning can shift practice patterns while improving outcomes and reducing costs. In Michigan, statewide payer–hospital quality collaboratives have shown that a “pay-for-participation” model can support sustained quality improvement infrastructure at scale, enabling data-driven practice change rather than isolated surgeon-level efforts [52,53,54]. Similarly, the Northern New England Cardiovascular Disease Study Group has demonstrated decades-long improvements in operative outcomes through a regional consortium model centered on transparent reporting and iterative process improvement [55]. These experiences support the feasibility of monitoring prosthesis sizing distributions, PPM rates, and selective AAE utilization as program-level indicators embedded in routine quality governance.

At the operational level, audit-and-feedback is one of the most evidence-supported strategies to improve professional practice, particularly when feedback is repeated, includes explicit targets, and is paired with an action plan [56]. Accordingly, if valve diameter optimization is advanced as a quality indicator, its implementation should explicitly incorporate periodic benchmarking dashboards (e.g., institutional PPM rates and small-valve implantation frequency), structured case review for “missed opportunities” for upsizing, and locally agreed thresholds that reflect patient risk and anatomy rather than a one-size-fits-all mandate [56]. This approach preserves clinical nuance while reducing unwarranted variation and ensuring that “upsizing” functions as a structured decision process aligned with lifetime management rather than a purely promotional metric.

## 8. Practical Considerations, Procedural Risk, and Training Implications

Despite encouraging contemporary safety data, aortic annular enlargement remains technically more complex than isolated SAVR. A final prerequisite for responsible adoption is workforce capability and operative safety. Educational evidence supports simulation, deliberate practice, and coaching as effective tools to improve technical performance, including for complex cardiac procedures. Simulation-based cardiac surgery curricula and deliberate practice models have been associated with measurable improvements in operative task performance and skill acquisition, supporting their use as adjuncts for disseminating technically demanding reconstructive procedures [57,58]. In parallel, structured surgical coaching has demonstrated benefits in operative skill development and may provide a practical mechanism for accelerating proficiency when introducing annular/root enlargement techniques into new programs [59]. Because AAE/ARE adds procedural steps and coordination demands, its implementation should be paired with mature intraoperative safety systems, such as checklist-driven workflow and team-training interventions, which have been associated with reductions in complications and mortality in broader surgical practice [60,61]. Together, these elements frame valve upsizing not as an isolated technical preference, but as a scalable quality strategy that requires measurement, feedback loops, and deliberate training infrastructure. Enlargement procedures require extended root dissection, patch reconstruction, and careful manipulation of the aortomitral curtain, which may increase cross-clamp duration and cardiopulmonary bypass time. In less experienced hands, potential risks include bleeding from patch suture lines, distortion of aortic root geometry, injury to adjacent structures such as the conduction system or mitral valve apparatus, and increased operative difficulty in heavily calcified or reoperative fields. These technical considerations underscore that enlargement is not a trivial extension of standard valve replacement but a reconstructive procedure that demands surgical expertise, appropriate case selection, and institutional support.

For any proposed quality metric to be clinically meaningful, it must remain grounded in the practical realities of surgical care delivery. Aortic annular enlargement, particularly when employing Y-incision and extended roof techniques, is inherently dependent on operator expertise and a detailed understanding of aortic root anatomy. As such, its successful implementation requires attention to both technical execution and institutional readiness.

Across published series, several recurring considerations have been identified. Adequate myocardial and cerebral protection, meticulous hemostasis, and careful handling of patch material are consistently emphasized as essential elements for minimizing bleeding, conduction disturbances, and other procedure-related complications [14,16,17,18,19,28,29]. Preoperative imaging also plays a central role in procedural planning. Comprehensive assessment using echocardiography and computed tomography enables accurate characterization of annular dimensions, root geometry, and coronary anatomy, facilitating informed decisions regarding the extent of enlargement and the anticipated geometry of the future ViV landing zone [13,15,31]. In addition, existing experience highlights the presence of a learning curve. While Y-incision enlargement is conceptually straightforward, consistent results depend on growing familiarity with patch sizing, suture technique, and aortotomy reconstruction within the operative team [14,18,19,30].

Historical concerns surrounding annular enlargement have centered on the potential for increased cross-clamp and cardiopulmonary bypass times, higher bleeding risk, and greater overall operative morbidity. Contemporary data suggest that these risks can be mitigated when procedures are performed in experienced centers with standardized techniques. Systematic reviews and comparative series indicate that incremental operative times associated with AAE are modest and that complication rates remain acceptable when procedures are performed in experienced centers [14,25,26,27,30]. For example, in the Michigan experience, Y-incision enlargement achieved substantially greater valve upsizing without a corresponding increase in operative mortality or major perioperative complications when compared with traditional enlargement techniques [14].

From an educational and workforce development standpoint, broader adoption of AAE is likely to depend on how effectively these techniques are incorporated into surgical training pathways. Integration of Y-incision and root enlargement methods into fellowship curricula, alongside simulation-based training and structured mentorship, may help normalize these approaches as part of the standard surgical armamentarium rather than as niche or exceptional procedures. As accumulating evidence and evolving guidelines increasingly emphasize lifetime management principles, annular enlargement and deliberate valve upsizing are poised to become core competencies for surgeons managing aortic valve disease [13,26,27,30].

## 9. Future Directions

Several areas remain incompletely defined and warrant focused investigation. First, an extended longitudinal follow-up of patients undergoing Y-incision annular enlargement is required to better characterize long-term prosthetic valve durability, patterns of left ventricular remodeling, and survival beyond five to ten years. While early and mid-term data are encouraging, longer-term outcomes will be essential to fully establish the lifetime impact of these reconstructive strategies. Second, the incorporation of computational fluid dynamics and patient-specific modeling into preoperative planning represents a promising avenue for refining valve sizing decisions. By integrating anatomical and hemodynamic simulations, future workflows may enable truly physiology-guided enlargement strategies, allowing surgeons to individualize the extent of annular expansion and prosthesis selection based on patient anatomy and projected lifetime treatment pathways [20,21,31]. Third, linkage of large-scale registry data capturing valve size, use of AAE, and ViV outcomes will help clarify the degree to which upsizing at the index operation influences the safety, durability, and effectiveness of subsequent transcatheter interventions. Recent analyses from large national registries have demonstrated the feasibility of integrating procedural detail with longitudinal outcome tracking to evaluate structural valve deterioration and reintervention patterns at scale, underscoring the potential for similar linkage strategies in annular enlargement research [62]. Furthermore, patient-specific computational modeling of transcatheter valve deployment and coronary flow dynamics has emerged as a promising tool for predicting post-procedural gradients and coronary obstruction risk prior to intervention [63]. In addition, emerging data suggest that CT-based assessment of sinus dimensions, coronary height, and virtual valve modeling can refine procedural planning and reduce the risk of coronary obstruction during redo interventions [64]. Integration of artificial intelligence–assisted imaging analytics may further enhance anatomical risk stratification and procedural prediction in structural heart disease [65]. As these technologies mature, integration of imaging-derived anatomical metrics with predictive simulation frameworks may enable more precise, physiology-guided enlargement and prosthesis selection strategies [66,67].

From a quality-improvement perspective, future metrics may extend beyond conventional measures such as prosthesis size distribution and prosthesis–patient mismatch (PPM) rates. Emerging “future-readiness” indicators, including predicted post-ViV gradients and coronary access feasibility derived from computed tomography and CFD simulations, could offer a more nuanced assessment of lifetime management quality. Such metrics may help distinguish between interventions that are merely adequate and those that optimally preserve long-term therapeutic flexibility.

## 10. Conclusions

Rather than reiterating established observations regarding prosthesis–patient mismatch, this review proposes a structured framework in which valve diameter optimization serves as a measurable and forward-looking determinant of lifetime therapeutic flexibility. Within contemporary lifetime management paradigms for aortic valve disease, prosthetic valve diameter appears to be an important determinant of long-term hemodynamic performance, risk of prosthesis–patient mismatch, feasibility of valve-in-valve interventions, and preservation of future treatment options. Accumulating clinical, imaging, and registry-based evidence challenges historical reluctance toward aortic annular enlargement, particularly when these procedures are performed in experienced centers with standardized techniques [14,25,26,27].

Advances in surgical reconstruction—most notably Y-incision annular enlargement with rectangular patch reconstruction and extended roof aortotomy closure—have expanded the achievable degree of annular and root enlargement to three to five valve sizes, fundamentally reshaping aortic root geometry in a manner favorable for both surgical and transcatheter therapies [14,15,16,17,18,19,20,28,29,30,31,32]. Computed tomography–based anatomical analyses and computational flow modeling consistently demonstrate the hemodynamic advantages of this approach, while statewide quality initiatives illustrate that systematic promotion of annular enlargement and valve upsizing can be implemented safely and effectively at scale [15,20,31,32,34]. Although much of the current evidence derives from observational and single-center experiences, the consistency of hemodynamic and clinical associations supports continued investigation and structured implementation within experienced programs.

For surgical programs committed to continuous quality improvement, deliberate optimization of valve diameter (through routine consideration of annular enlargement, reduction in PPM, and alignment of prosthesis internal diameter with CT-derived annular dimensions) may serve as a candidate performance metric within appropriately selected patient populations. In practical terms, this reframes the decision-making process at the time of SAVR: the operative goal is no longer simply to implant a valve, but to implant one that will serve the patient effectively across the full trajectory of their disease.

## Figures and Tables

**Table 1:** Impact of Prosthetic Valve Diameter on Hemodynamics, Reintervention Risk, and Lifetime Management.

Clinical Domain	Consequences Associated with Small Prosthetic Valves (≤23 mm)	Consequences Associated with Upsized Prosthetic Valves (≥25 mm)	Key Supporting Evidence
Early Hemodynamic Performance	Persistently elevated mean gradients, reduced effective orifice area, early prosthesis–patient mismatch, and attenuated left ventricular reverse remodeling	Lower transvalvular gradients, larger effective orifice area, and more robust left ventricular reverse remodeling	Pibarot & Dumesnil [4]; Head et al. [6]; Fallon et al. [7]
Risk of Prosthesis–Patient Mismatch (PPM)	High prevalence of moderate and severe PPM	Marked reduction or near-elimination of clinically significant PPM	Meta-analyses and registry data [4,9,11]
Durability of Surgical Prosthesis	Increased transvalvular stress and unfavorable shear conditions that may accelerate structural valve degeneration	More favorable flow and shear profiles associated with improved prosthetic durability	Computational modeling [14,15]; clinical series [16,17,18]
Valve-in-Valve (ViV) TAVR Feasibility	Elevated post-ViV gradients, limited valve expansion, increased risk of coronary obstruction and sinus sequestration	Acceptable post-ViV gradients with expanded root geometry and reduced coronary risk	CT-based morphology studies [19]; ViV hemodynamic analyses [14,15]
Future Coronary Access	Coronary access often challenging or unsafe, particularly after ViV implantation	Preserved coronary clearance due to enlarged sinus and sinotubular junction geometry	CT-based studies [14,19]
Redo-SAVR Complexity	Increased technical complexity due to small root geometry and prior prosthesis constraints	More favorable anatomy facilitating safer and more straightforward redo procedures	Reoperative series [17,20]
Lifetime Management Trajectory	Constrained therapeutic options, higher likelihood of recurrent heart failure and complex reinterventions	Expanded lifetime options, improved ViV feasibility, and reduced long-term heart failure burden	Lifetime management frameworks [8,21]

Interpretation: Prosthetic valve diameter is not a neutral operative variable; it influences postoperative hemodynamics, durability, reintervention strategies, and long-term disease trajectory. Abbreviations: EOA, effective orifice area; HF, heart failure; LV, left ventricle/left ventricular; PPM, prosthesis–patient mismatch; SAVR, surgical aortic valve replacement; STJ, sinotubular junction; TAVR, transcatheter aortic valve replacement; ViV, valve-in-valve.

**Table 2:** Anatomic and Hemodynamic Remodeling Achieved with Y-Incision and Roof AAE.

Remodeling Parameter	Magnitude of Change with Y-Incision/Roof AAE	Functional and Clinical Implications	Evidence Source
Annular (Basal Ring) Diameter	Enlargement equivalent to +3 to +5 valve sizes	Enables implantation of large prostheses and minimizes risk of prosthesis–patient mismatch	Yang series [16,17,26,27,28,29]
Sinus of Valsalva Dimensions (LCA/NCC/RCC)	Increase of approximately 4–8 mm	Improved coronary clearance and reduced risk of sinus sequestration	Truesdell et al. [19]
Sinotubular Junction Diameter	Increase of approximately 6–10 mm	Creation of a more favorable landing zone for valve-in-valve TAVR and reduced supravalvular obstruction	Roof technique CT analyses [14,19]
Left Ventricular Outflow Tract Geometry	Transition toward a more cylindrical outflow configuration	Reduced flow turbulence and improved systolic ejection efficiency	Yang et al. [16,20]
Peak Systolic Velocity	Reduction of 30–55% in computational fluid dynamics simulations	Lower shear stress and improved prosthetic flow environment	Bonini et al. [15]
Transvalvular Pressure Gradient	Reduction of 80–92% in CFD-based valve-in-valve models	Substantial improvement in post–valve-in-valve hemodynamics	Bonini et al. [15]
Turbulent Kinetic Energy	Marked reduction	Decreased energy loss and optimized left ventricular workload	Ghimire modeling [31]
Paravalvular Leak Risk	Effectively eliminated with appropriate patch reconstruction	Improved early outcomes and reduced need for reintervention	Yang; Yazdchi; Monaghan [16,17,20]

Interpretation: Y-incision and roof enlargement techniques result in comprehensive root remodeling involving the annulus, sinuses, and sinotubular junction, generating anatomy that is hemodynamically favorable for both surgical and transcatheter valve therapies. Abbreviations: AAE, aortic annular enlargement; CFD, computational fluid dynamics; EOA, effective orifice area; LCA, left coronary cusp; NCC, non-coronary cusp; RCC, right coronary cusp; LVOT, left ventricular outflow tract; PPM, prosthesis–patient mismatch; PSV, peak systolic velocity; STJ, sinotubular junction; ViV, valve-in-valve.

**Table 3:** Comparative Technical and Geometric Characteristics of Aortic Annular Enlargement Techniques.

Feature	Nicks Technique	Manouguian Technique	Y-Incision Technique	Y-Incision + Roof Extension
Primary Incision Location	Posterior through non-coronary sinus	Posterior extending into aortomitral curtain	Y-shaped incision across aortomitral curtain	Y-incision with additional superior roof extension
Direction of Enlargement	Posterior only	Posterior + partial curtain	Symmetric basal ring expansion	Basal ring + sinotubular junction expansion
Typical Prosthesis Upsizing	+1–2 valve sizes	+1–2 valve sizes	+3–4 valve sizes	+3–5 valve sizes
Sinotubular Junction (STJ) Remodeling	Minimal	Minimal	Limited	Significant and deliberate
Root Geometry Effect	Localized posterior dilation	Partial posterior enlargement	Circumferential annular widening	Comprehensive annular and supravalvular remodeling
Risk of Mitral Distortion	Low	Moderate (curtain involvement)	Low (rectangular patch preserves geometry)	Low
Suitability for Future ViV	Limited by residual STJ constraint	Moderate	High	Very high
Technical Complexity	Moderate	Moderate	Advanced	Advanced

**Table 4:** Metrics for Evaluating Valve Diameter Optimization Programs.

Evaluation Metric	Operational Definition	Suggested Benchmark	Rationale
Index Prosthesis Size Appropriateness	Proportion of SAVR cases in which prosthesis internal diameter is within ≤1–2 mm of CT-derived annular size	>80% of SAVR cases (annulus ≤25 mm)	Promotes optimal hemodynamics and valve-in-valve feasibility
Utilization of Annular Enlargement (AAE Rate)	Percentage of SAVR cases employing AAE in patients at moderate or high risk of prosthesis–patient mismatch	20–30% (statewide reference benchmark)	Reduces PPM prevalence and improves valve size distribution
Postoperative PPM Incidence	Rate of moderate or severe prosthesis–patient mismatch assessed by indexed effective orifice area at 30 days	<10% moderate; <2% severe	Clinically meaningful patient-centered outcome
Root Geometry Optimization	CT-confirmed expansion of annulus, sinuses, and sinotubular junction following annular enlargement	Annulus ≥23–25 mm with sinus/STJ enlargement	Aligns reconstructed anatomy with future valve-in-valve requirements
Valve-in-Valve Readiness Index	Predicted feasibility of future valve-in-valve TAVR with acceptable gradients and coronary access	≥85% feasibility projection	Integrates anatomy, prosthesis size, and enlargement strategy
Surgeon-Level Valve Size Distribution	Median prosthesis size implanted by individual surgeons	Median ≥ 25 mm	Identifies unwarranted practice variation
Long-Term Outcome Tracking	Heart failure readmissions, post–valve-in-valve gradients, redo-SAVR rates	Progressive year-over-year reduction	Captures lifetime benefit of optimized index surgery

Interpretation: Valve upsizing is measurable, auditable, and directly linked to patient-centered outcomes across the lifetime of aortic valve disease. Abbreviations: AAE, aortic annular enlargement; CT, computed tomography; HF, heart failure; iEOA, indexed effective orifice area; PPM, prosthesis–patient mismatch; QI, quality improvement; SAVR, surgical aortic valve replacement; STJ, sinotubular junction; ViV, valve-in-valve.

## Data Availability

The data that support the findings of this study are available from the corresponding author upon reasonable request.
